# A Life Cycle Perspective to Assess the Environmental and Economic Impacts of Innovative Technologies in Extra Virgin Olive Oil Extraction

**DOI:** 10.3390/foods8060209

**Published:** 2019-06-13

**Authors:** Teodora Stillitano, Giacomo Falcone, Anna Irene De Luca, Antonio Piga, Paola Conte, Alfio Strano, Giovanni Gulisano

**Affiliations:** 1 Department of Agriculture, Mediterranean University of Reggio Calabria, Feo di Vito, 89122 Reggio Calabria, Italy; teodora.stillitano@unirc.it (T.S.); giacomo.falcone@unirc.it (G.F.); astrano@unirc.it (A.S.); ggulisano@unirc.it (G.G.); 2 Department of Agriculture, University of Sassari, Viale Italia 39/A, 07100 Sassari, Italy; pigaa@uniss.it (A.P.); pconte@uniss.it (P.C.)

**Keywords:** extra virgin olive oil (EVOO), life cycle assessment, life cycle costing, olive-oil industry, processing innovation, malaxation

## Abstract

Advances in the adoption of technological innovations represent a great driver to improve the competitiveness of the Italian extra virgin olive oil (EVOO) industry. This work assesses the efficiency of an innovative extraction plant (with low oxidative impact, heating of paste before malaxation and a special decanter that avoids the final vertical centrifugation) in terms of oil yield and quality, and economic and environmental impacts. Economic and environmental impacts were evaluated by using both life cycle costing and life cycle assessment methodologies. A sensitivity analysis was also performed to highlight the uncertain factors that may strongly affect the results. Findings showed that olive milling with the innovative plant resulted in olive oil with a significant increase in quality, although the extraction yield was significantly higher when using conventional technology. In terms of environmental results, an average growth of 4.5% of the impacts in all categories was reached. The economic results revealed the highest extraction cost for the innovative scenario as well as the lower profitability, although a positive return in investment feasibility can be achieved due to an increase in the olive oil selling price. These findings could be useful to highlight the main hotspots in EVOO production and to suggest improvements for more sustainable management.

## 1. Introduction

As it is now widely recognized, olive oil occupies a central role in Mediterranean eating habits, both due to culinary heritages and, especially in the last decades, to increasing awareness towards healthy concerns [1]. Furthermore, it is equally known as olive farms and oil mills, for most of the areas facing the Mediterranean basin, represent important institutions for several goals of collective interest for example, the retention of traditions, the environmental safeguard, the support to the local economy [2]. Today increasingly, agribusiness is up against crucial challenges carried by an unstable global scenery, marked by changes in ecological balances, demographic transitions, and consumption dynamics. From the point of view of farms, producers, and operators of the olive sector, meeting those challenges is the most important key to obtain a sustainable competitive advantage. This is particularly true for quality food products, such as for extra virgin olive oil (EVOO), which is crucial to develop effective management strategies, in order to satisfy consumers’ preferences, by optimizing the profitability conditions for the long-term success of the companies. From this point of view, and for the future development of the whole olive oil sector, the producers’ needs linked to the requirements to compete on the current market cannot be neglected. In this sense, the adoption of technological innovations, as competitive advantage factor, can be a key element to productivity improvements, especially for quality attributes, and then for obtaining greater financial incomes, but in the meantime, for adapting the agri-food processes to societal claims. In the recent narrative of agri-food systems, different pathways and paradigms towards increasing sustainability levels are considered [3]. For the majority, it means to limit resource loads and environmental damage, while also lowering production costs and/or providing ‘higher quality’ products. However, several authors reported that the processes of adoption of technological innovations in the Italian EVOO industry have always been delayed and controversial [4,5].

Based on these premises, and since the current loss in competitiveness recorded by the Italian olive-oil industry [6] the main objective of this work is to evaluate, from an economic and environmental point of view, the introduction of an innovative system with low oxidative impact for EVOO extraction and its effects on oil yield and quality and plant energy consumption, and its implications in revenues terms for olive entrepreneurs.

### 1.1. Innovative EVOO Extraction Technologies

EVOO quality is strictly dependent on several factors, such as cultivar, agronomic practices, and olive oil extraction technology [7]. Processing of olives to obtain EVOO is carried out solely by mechanical operations, which have been strongly improved during the last thirty years [8,9,10,11,12]. Malaxation, which involves mixing of the crushed olive paste to promote oil drop coalescence, has prominent importance over the whole extraction process. The main proposed improvements of malaxation in recent years have been the use of vertical malaxators working at low oxygen pressure [13,14] and the heating of crushed olive paste before entering the malaxators by tubular heat exchanger [15,16] or by microwave [17,18]. These new technologies result in reducing paste oxidation and converting malaxation from a discontinuous to a continuous process, thus improving the quality of olive oils.

The literature cited above refers to only one of the technologies being used at the same time, thus it would be helpful to study the effect of both (tubular heat exchanger and low oxygen malaxator) followed by a further improvement in the centrifugation phase accomplished by a special decanter that excludes the use of vertical centrifugation.

### 1.2. Life Cycle Analysis

The application of life cycle methodologies is recognized as very effective to measure the impacts of a product/service because it allows the consideration of an exhaustive range of components within all the phases of its production process. The real life cycle of the object under study is analyzed considering its interaction with the environmental context, in terms of raw materials, extraction, or the addition of substances, land use, etc., as well with the economic dimensions, as production costs, costs related to implementing technology, revenues, cash-flows, etc. According to the International Organization for Standardization (ISO) [19,20] a correct use of the life cycle assessment (LCA), for measurement of environmental loads, establishes to take into account specific categories of impact. In particular, ISO norms distinguish four different steps: Definition of the goal and scope of the study, including a clear statement on the specification of the functional unit (FU)—i.e., the measurement unit to which all inputs and outputs data are related—as well as of system boundaries, description of data quality, and procedures of allocation. The construction of the life cycle inventory (LCI), with qualitative and quantitative data collection, calculation of incoming and outgoing flows (energy, materials, and emissions), and validation. The life cycle inventory assessment (LCIA) that represents the third step and consists of quantifying potential environmental impacts, through the selection of impact categories and, for all of them, relevant indicators and characterization models. The final phase is the interpretation of results highlights the hotspots of the life cycle analyzed and allows formulating suggestions to improve the production process. This framework was adopted for investigating particular aspects of environmental sustainability, as for example LCA-based like the carbon footprint [21], water footprint [22], ecological footprint [23], and environmental footprint of products (PEF) and organization (OEF) [24]. Frequently, LCA methodology is applied to the agri-food sector [25] representing until today a field of study strongly investigated, due to the critical issues represented by the mixture of ecological constraints, anthropic activities, and industrial processes.

From the economic point of view, the life cycle analysis, properly conducted by means of life cycle costing (LCC) approaches, allows us to consider both the initial and operating costs incurred throughout the entire life cycle of the product or system. This method through a punctual costs assessment suggests an improvement for an optimal budget allocation during the system’s/product’s lifetime, as well as better business performance [26,27]. Several methods [28,29,30] and standards [31,32,33] for performing and harmonizing LCC were developed over time, but today again a standard LCC methodology does not occur; the most widespread approach is the discounting technique and cash flows models [34,35]. Almost all LCC applications to agri-food processes uses the so-called “Conventional LCC”, which assesses only internal costs along the life cycle of a product, within the economic system.

Olive oil is one of the most studied agri-food products by scholars and practitioners of the life cycle analyses. Particularly, among the most relevant studies recently performed, have highlighted the following: Ten applications related to an environmental assessment by means of LCA [36,37,38,39,40,41,42,43,44,45]; four studies on LCC analysis [2,46,47,48]; and six works with integrated application of LCA and LCC [49,50,51,52,53,54].

The purpose of this work is to quantify the environmental loads and economic implications in the adoption of the innovative EVOO extraction system. Data input useful to conduct LCA and LCC analyses was provided by experimental trials carried out in Sardinia (Italy). In terms of environmental impacts, the following categories were evaluated: Global warming, depletion of the ozone layer, eutrophication, acidification, human and ecosystem toxicity, depletion of natural resources, energy consumption, land use, and water use. Relatively to the economic affordability, specific cost items were accounted by monetizing inputs and outputs values, as well as fixed and variable costs incurred in the production system. Obtained results allowed verifying the environmental and economic sustainability of the experimental technology for EVOO extraction, in order to highlight the main hotspots of the industrial process and suggest potential improvements for more sustainable management.

## 2. Materials and Methods 

### 2.1. Experimentation Description

#### 2.1.1. Plant Material

**Figure 1:**
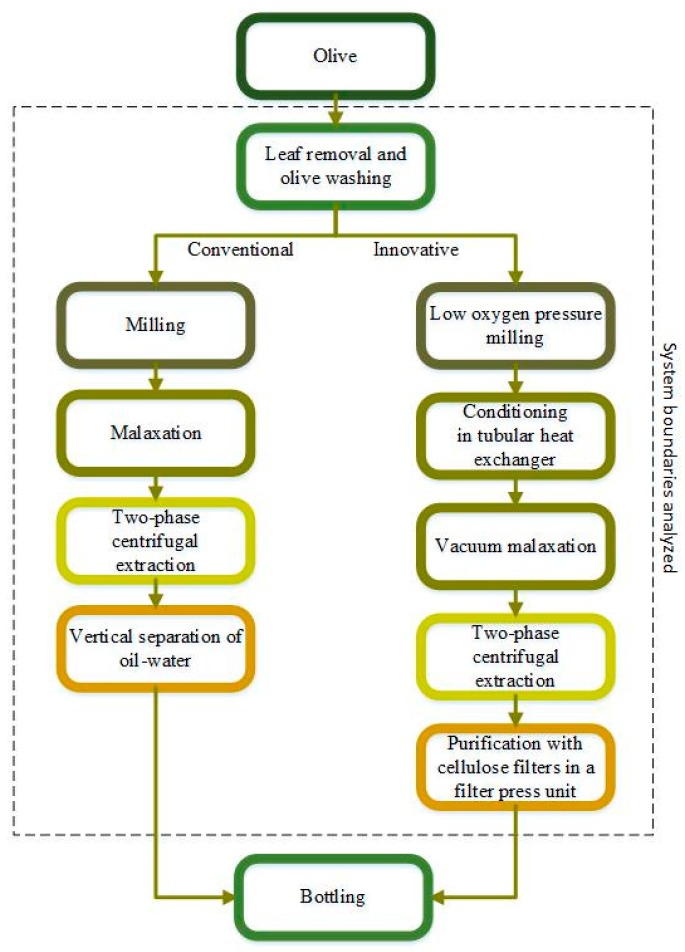
System boundaries of the olive oil milling plant.

Homogeneous olive samples of 600 kg of cultivar Bosana were mechanically harvested in November 2017 at an optimal stage of ripening (60% just turned dark-colored and the 40% green). Samples were divided into two 300 kg batches and milled within three hours with two separate mills, one using conventional technology and the other one with an innovative approach, as illustrated in Figure 1.

#### 2.1.2. Virgin Olive Oil Processing

The first batch of olives was milled in a plant at Olio Corax-Azienda Agricola di Francesco Piras located in Alghero (Sardinia-Italy), with a conventional plant (CONV). Olives were de-leafed (Mod. Imperatrice, Mercuri, Melicucco (RC), Italy), washed (Mod. Optima L20, Pieralisi MAIP Spa, Jesi (AN), Italy), and milled with a continuous Pieralisi MAIP Spa (Pieralisi MAIP Spa, Jesi (AN), Italy) with a theoretical work capability of 2500 kg·h^−1^ plant composed of hummer/blade crusher working at 2800 rpm (Mod. Fp.2800 rpm) with a theoretical work capability of 3000 kg·h^−1^ followed by a malaxer machine (Mod. Pieralisi V3, Pieralisi MAIP Spa) with a work capability of 4000 kg·h^−1^. The paste obtained was malaxed for 20 min at 25 °C, after that was sent with a flow rate of 1800 kg·h^−1^ to a two-phase horizontal decanter (Mod. M3, Pieralisi MAIP Spa) with a work capability of 2000 kg·h^−1^ and a liquid/liquid vertical centrifuge (Mod. Plutone, Pieralisi MAIP Spa) with a work capability of 1500/1800 kg·h^−1^. Paste entering the decanter was diluted with 30% water.

The extraction of the second batch was carried out at Su Molinu mill located in Oliena (Sardinia-Italy), with an innovative Mori Tem plant (Tavernelle Val di Pesa (FI), Italy; INN). Olives were de-leafed (Mod. EN_300_L2) washed (Mod. DLE SUPER_T Lava e asciuga) and sent to a blade crusher Mori Tem plant (Tavernelle Val di Pesa, FI, Italy) working at low oxygen pressure (Mod. FR 350, Mori Tem plant) with a theoretical work capability of 2000 kg·h^−1^. The paste obtained was conditioned at 25 °C in a tubular heat exchanger (Mod. ST4, Mori Tem plant) with a work capability of 1000 kg·h^−1^ and sent to a malaxer machine (Mod. Gramola cultivar, Mori Tem plant) with a work capability of 400 kg·h^−1^ where it was malaxed for 20 min at 25 °C under reduced ambient pressure (0.2 atm). The paste enters the vacuum malaxator through the bottom side and is malaxed by a vertical rotor fitted with blades. The malaxed paste was then centrifuged, without water addition, with an innovative two-phase centrifugation system (Mod. TL 1000, Mori Tem plant) with a work capability of 1000 kg·h^−1^ followed by purification with cellulose filters in a filter press unit (EUR 40, Mori Tem plant) with a work capability of 600 kg·h^−1^, thus avoiding the use of a vertical centrifuge.

The extraction yield (Y) was the percentage ratio between the amount of oil obtained and that of the olives milled.

#### 2.1.3. Oil Bottling, Storing, and Sampling

The oils obtained were immediately bottled in 250 mL dark glass bottles with an automatic bottling machine leaving an air headspace was 27.2 mm. Bottled samples were stored in the dark in a chamber at 20 °C and analyzed within one week.

### 2.2. Legal Quality Indices, Chlorophylls, Polyphenols, and Tocopherols

Acidity (% oleic acid) and peroxide values (meq O_2_ kg^−1^) were determined by the methods reported in Regulation EEC/2568/91 of the European Union Commission. Chlorophylls were evaluated according to the spectrophotometric methods proposed by Pokorny et al. [55]. The instrument wavelengths (mod. 8453, Hewlett-Packard, Palo Alto, CA, USA) were set at 630, 670, and 710 nm and data were expressed as mg kg^−1^ of pheophytin A. Total polyphenols were extracted according to the method proposed by Pirisi et al. [56]. The extract was subjected to spectrophotometric determination of total polyphenols and following Singleton and Rossi [57]. The instrument wavelength (mod. 8453, Hewlett-Packard) was set at 750 nm and data were expressed as mg gallic acid kg^−1^ oil. Tocopherols were detected and quantified by the method proposed by Gimeno et al. [8], using an HPLC Agilent 1100 Series (Agilent Technologies, Palo Alto, CA, USA), detector FLD Agilent 1100 Series set at 290 nm for excitation and 330 nm for emission, a Gemini C18 100 × 4.6 mm ID, 3 μm particle size column (Phenomenex, Torrence, CA, USA), a mobile phase of methanol-water (98:2 *v/v*) and a flow rate of 2 mL/min. Results were expressed as mg of α-tocopherol per kg of oil by comparison of the chromatographic area with a standard response curve. The antioxidant activity was determined on the polyphenol extract with the DPPH^●^ stable radical, according to the method proposed by Ramadan and Morsel. [58]. In particular, 50 μg of methanolic extract was dissolved in 500 μL of toluene, the mixture vortexed for 1 min, and an aliquot of 200 μL made reacted in a 1 cm path cuvette at 22 °C under continuous stirring with 1.8 mL of a 10^−4^ M toluene solution of DPPH^●^. The decrease in absorbance at 515 nm was recorded at 1, 5, 15, 30, and 60 min against a blank of pure toluene using a spectrophotometer (mod. 8453, Hewlett-Packard). The antioxidant activity was considered as the difference in absorbance between each sample and a toluene DPPH^●^ solution without the sample (control) and calculated as percentage of inhibition with the following equation:

% of inhibition = (1 − (Asample/Acontrol)) × 100.(1)

The concentration of the sample that results in a 50% inhibition (EC50) was then calculated.

### 2.3. Statistical Analysis

All determinations were done in triplicate. Data were evaluated by a one-way analysis of variance using Statistica 6.0 for Windows (StatSoft, Inc., Tulsa, OK, USA ). Extraction technology was chosen as the variable. Means, where appropriate, were separated by Tukey’s test for *p* ≤ 0.01.

### 2.4. LCA and LCC Implementation

To account for the environmental and economic burdens of the olive oil extraction systems under study, LCA and LCC approaches were adopted. These are widely accepted methodologies in the field of sustainability evaluation due to their approach, which considers the impacts of a product from raw material extraction to production, use, and disposal. LCA was performed following the framework suggested by ISO 14040:2006 [19] and 14044:2006 [20] and considering four different methodological steps: Goal and scope definition; life cycle inventory; life cycle impact assessment (LCIA); and interpretation. In this work, the LCC methodological approach of Ciroth et al. [59] and Moreau and Weidema [60] was conducted. This approach, based on a cash flow model borrows the same computational framework of LCA. In this way, the system boundary and the functional unit were similar to those of the LCA. As argued by Kloepffer [61] and Swarr et al. [62] the LCC method only includes real money flows (costs and revenues) of each unit process, in order to avoid double accounting between LCA and LCC.

#### 2.4.1. Goal and Scope Definition

The study is aimed at analyzing the environmental and economic impact of an innovative olive oil milling plant designed to enhance the quality of EVO oil. The environmental and economic profile of an innovative (INN) scenario was compared with a conventional (CONV) olive oil milling plant, in order to highlight the advantages or drawbacks of the innovative plant.

#### 2.4.2. Functional Unit and System Boundaries

One liter (1 L) of extra virgin olive oil was adopted as a shared functional unit (FU) for both LCA and LCC analyses performed in this work. Both olive oil production systems under study, i.e., CONV and INN, embedded the olive oil extraction whereas the olive production, the olive oil bottling and packaging, the distribution to the consumers, and the end-use were not included within the system boundaries. Therefore, an analysis from farm’s gate to gate of olive oil milling plant was followed and all the unit processes associated with the olive oil extraction were identified (Figure 1). In the CONV scenario, the following processes were taken into account: (i) Leaf removal and olive washing, (ii) milling, (iii) malaxation, (iv) two-phase centrifugal extraction, and (v) vertical separation of oil-water. In the INN scenario, they were: (i) Leaf removal and olive washing, (ii) low oxygen pressure milling, (iii) conditioning in the tubular heat exchanger, (iv) vacuum malaxation, (v) two-phase centrifugation extraction, and (vi) purification with cellulose filters in a filter press unit.

#### 2.4.3. Environmental Analysis

Data of the core process analyzed were directly measured in two milling plants located in Sardinia. In particular, data on electricity, water, and electricity for heat consumption were collected by means of a specific survey form, as well as data on olive oil yield and waste and co-product production (Table 1). Data related to electricity and heat production, water source, and waste treatment were taken from secondary sources, particularly from Ecoinvent 3.4 and Agri-footprint 4.0 databases.

Input and output were allocated considering the economic value of EVOO and co-products (pomace) [63].

Input and output collected in the life cycle inventory were processed through Simapro 8.5 software (PRé Consultants BV, Stationsplein Amersfoort, Netherlands) by using the ILCD 2011 midpoint method (Table 2). Ionizing radiation and land use indicators were excluded because the olive oil milling process does not have impacts in these categories.

A sensitivity analysis was carried out in order to define the influence of the oil yield on the eco-profile of the innovative olive oil process. In particular, an increase and a decrease in the yield of the extraction equal to ±3% were considered and results were compared to the innovative process with the actual yield and conventional scenario. Additional analyses were performed by expressing the results in function of the quality of oil, the element that represents the main purpose of the experiments. In this way, results were expressed by changing the functional unit from 1 L of EVOO to 1 mg of chlorophyll, total polyphenols, and total tocopherols quality parameters.

#### 2.4.4. Economic Analysis

To carry on with the economic elaborations, an average lifespan for the olive oil milling plant was assumed to an equal extent of 20 years. All of the costs occurring throughout the life cycle of each scenario (CONV and INN) were considered. These costs were calculated through the monetization of all the collected inputs and outputs, i.e., by multiplying the measured quantities by its unit prices [64]. Particularly, costs related to the extraction plant investment (start-up costs), operating costs of the extraction phase, and disposal costs (end of life costs) were analyzed.

Regarding the start-up costs, the building component and extraction plant, in terms of investment, were considered. The building component consisted of a wide shed with a surface of about 500 m^2^, able to contain the machinery for the extraction process and storage tank. The extraction plants have been illustrated in the virgin olive oil processing section.

To determine the total cost of 1 L of EVOO, operating costs were split in variable and fixed costs. The variable costs included the input costs for olive oil extraction (for example, electricity consumption by machinery), human labor cost, and interests on advance capital. Within the fixed costs, machinery and land investments ownership costs (i.e., the depreciation, insurance, and maintenance), land rent, interests on capital goods, taxes, and administration overheads were accounted for [65]. In this study, the input cost was determined according to the current market prices (i.e., 2017), while the human labor cost was estimated according to the local current wage. To estimate the rental cost for land use, average local rental prices were considered. The interests in advance capital and capital goods were determined by applying an interest rate of 4.5% and 2%, respectively. The administrative overheads were estimated to be 5% of the gross production value, which corresponds to the annual total revenues [2]. The total revenues were calculated by multiplying the olive oil yield for its market price. As the olive oil selling price is strongly influenced by the alternate bearing of olive trees, climatic conditions and plant diseases, which affects the olive oil yield [66], we collected the prices of EVO oil in the Italian market by the Istituto di Servizi per il Mercato agricolo Alimentare and referred to the 2016/2017 and 2017/2018 harvesting seasons. Then an average price equal to 5.40 € L^−1^ was assumed. In the comparison analysis between INN and CONV scenarios, we have not considered the potential premium price of oil extracted by the innovative plant due to the improvement of the quality also linked to the increase in polyphenols and then to the highest health value of the product [4].

Concerning the end of life costs, the plant disposal for extraction component was estimated as the residual value of the machinery and calculated at 50% of the cost of construction in 2017 [67].

In the second step of the analysis, costs and revenues were discounted for the entire life cycle of 20 years (*n*), using a discount rate (*r*) of 2% attributed based on the opportunity cost approach in terms of alternative investments with similar risk and time. In particular, the discount rate refers to the average return rate of Italian government bonds in 2017. In this way, the cash flows generated at different stages of the project lifespan were assessed. Throughout the life cycle, we supposed constant prices, and therefore, we could exclude adjustments for inflation [68].

In Table 3, the most important technical parameters needed to carry out economic analysis are illustrated.

In this work, the LCC is implemented in conjunction with specific economic indicators to evaluate the profitability and investment feasibility of the olive oil production systems under study. In particular, to conduct financial analysis three specific indicators were identified, i.e., discounted gross margin (DGM), net present value (NPV), and discounted life cycle costs (DLCC), as illustrated in Table 4. 

The DGM indicator was calculated by subtracting the discounted variable costs (VC) from the discounted total revenues (TR), providing information on project profitability. Regarding the NPV indicator, this represents the sum of discounted future cash flows incurred during the whole life cycle and was chosen as an indicator of the investment feasibility [69,70,71]. The DLCC calculation was made by summing up the investment costs (IC), the present value of the operating costs (OP) of each process, and discounted disposal costs (DC); this indicator has been applied to estimate the overall cost of the different scenarios, with the purpose of recognizing the primary processes responsible for most of the economic impacts [53]. Finally, each indicator value was divided by the total EVOO production (TP).

## 3. Results

Table 5 reports data on yield (Y) and chemical parameters obtained with the two extraction technologies. Although the Y was significantly higher when using the CONV technology, olive milling with the INN plant resulted in olive oil with a significant increase in quality (Table 5). Both technologies gave extra virgin olive oils, but the INN samples revealed a significantly lower peroxide content, with respect to CONV samples, surely due to the lower oxidative stress exerted by this technology. Moreover, chlorophylls, total polyphenols, and tocopherols content were significantly increased by the INN plant as already reported [14]. As a consequence, the antioxidant activity of INN oils was significantly improved, with respect to CONV samples.

### 3.1. Environmental Results

Analysis of results shows an increase of impacts on average of 4.5% for all impact categories. The rise of impacts can be due to the lower extraction yield of the INN milling plant (Table 6).

**Figure 2:**
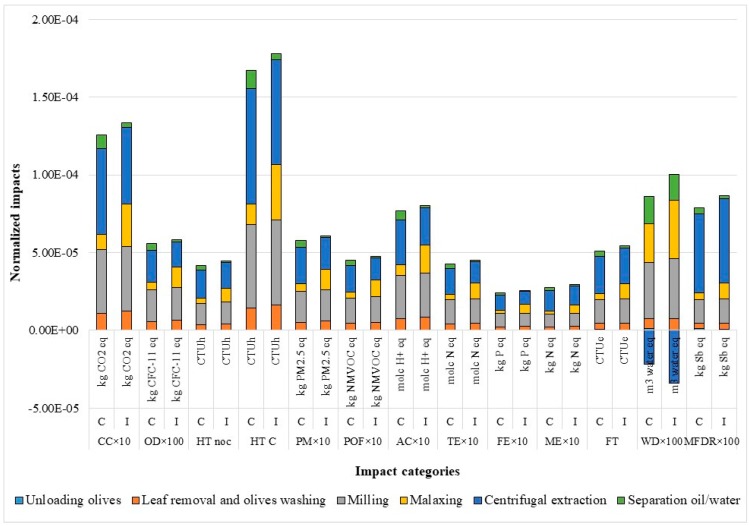
Hotspot analysis of conventional (C) and innovative (I) scenarios. CC: Climate change; OD: Ozone depletion; HT noc: Human toxicity, non cancer effects; HTC: Human toxicity, cancer effects; PM: Particulate matter; POF: Photochemical ozone formation; AC: Acidification; TE: Terrestrial eutrophication; FE: Freshwater eutrophication; ME: Marine eutrophication; FT: Freshwater ecotoxicity; WD: Water resource depletion; and MFDR: Mineral, fossil, and ren resource depletion.

Climate change impacts represent about 4% of total impacts and they are mainly caused by the consumption of electricity during the milling and centrifugal extraction operations. The use of this input in processing phases causes the largest share of impacts in the following categories: Human toxicity, non-cancer effects for 13.7% of total impact; and human toxicity, cancer effects, for 54.5% of the total impact. Same trends are shown for other impact categories analyzed that represent the following share of the total impact: Ozone depletion 0.2%; particulate matter 1.9%; photochemical ozone formation 1.5%; acidification 2.5%; terrestrial eutrophication 1.4%; freshwater eutrophication 0.8%; marine eutrophication 0.9%; freshwater ecotoxicity 16.4%; mineral, fossil, and ren resource depletion 0.3%. The impact linked to water resource depletion (2.1%) is caused by process water used for the washing of olives and for the fluidification of the olive mixture during the malaxing and by the washing of the plant at the end of milling operations. Positive impacts are generated by the treatment of wastewater, operation attached to the centrifugal extraction (Figure 2).

**Figure 3:**
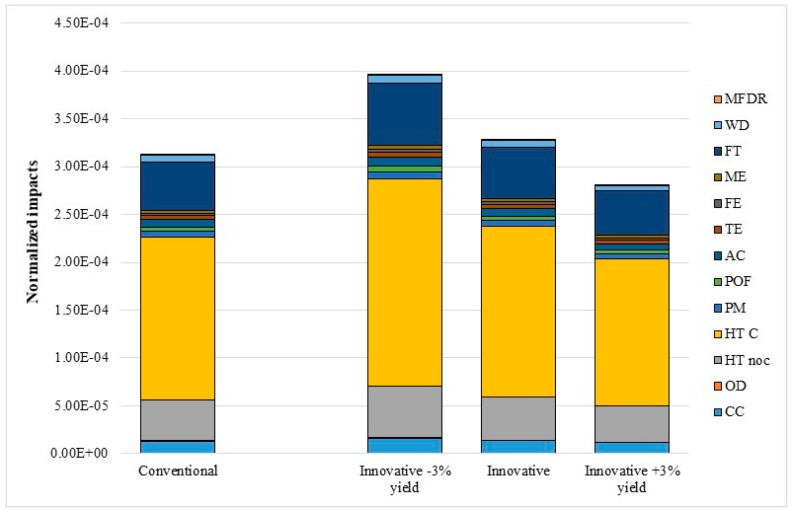
Sensitivity analysis. CC: Climate change; OD: Ozone depletion; HT noc: Human toxicity, non cancer effects; HTC: Human toxicity, cancer effects; PM: Particulate matter; POF: Photochemical ozone formation; AC: Acidification; TE: Terrestrial eutrophication; FE: Freshwater eutrophication; ME: Marine eutrophication; FT: Freshwater ecotoxicity; WD: Water resource depletion; and MFDR: Mineral, fossil, and ren resource depletion.

The sensitivity analysis shows that the extraction yield plays a fundamental role in the eco-profile of the innovative scenario, allowing a linear reduction of impacts in all categories considered (Figure 3).

**Figure 4:**
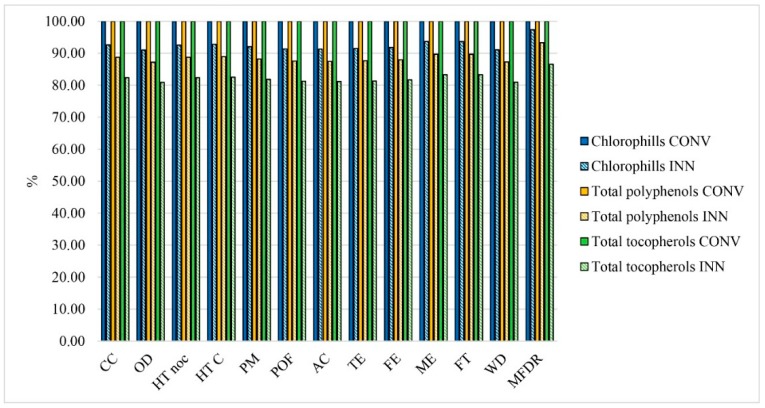
Comparative assessment in the function of 1 mg of quality parameters. CC: Climate change; OD: Ozone depletion; HT noc: Human toxicity, non cancer effects; HTC: Human toxicity, cancer effects; PM: Particulate matter; POF: Photochemical ozone formation; AC: Acidification; TE: Terrestrial eutrophication; FE: Freshwater eutrophication; ME: Marine eutrophication; FT: Freshwater ecotoxicity; WD: Water resource depletion; and MFDR: Mineral, fossil, and ren resource depletion.

Changing the perspective of results in the function of enhancement of the quality of olive oil, data were processed by using as FU 1 mg of quality parameters (1 mg of chlorophylls, 1 mg of total polyphenols, and 1 mg of total tocopherols). This simulation shows that the reduction of the yield in oil extraction is comprehensively compensated by quality enhancement, which allows extracting, despite the lower olive oil yield, a larger quantity of substances favorable for the quality of the olive oil (Figure 4).

### 3.2. Economic Results

The different EVOO extraction technology employed in the CONV and INN plant caused changes in productivity, as well as in the total production cost and profitability of the systems evaluated. It is necessary to specify that the economic results strictly depended on the production yield, which was higher in the conventional plant than the innovative one.

**Figure 5:**
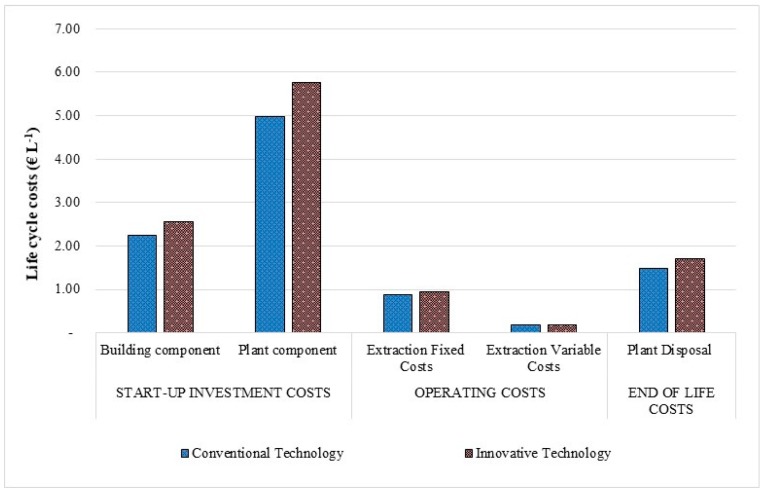
Life cycle costs occurring in the olive oil extraction phase.

Figure 5 shows, in terms of FU, the total life cycle cost of each scenario divided into costs related to start-up, operating, and end of life. The highest LCC of 9.77 € L^−1^ is estimated for the INN plant, which has 14% larger LCC compared to the CONV one. The greatest contributors are the start-up investment costs, ranging from 74.48% for the INN system to 73.90% for the CONV one. Plant component, which corresponds to the olive oil extraction machinery, contributes overall 51.56% and 50.94% to the costs, respectively.

The costs related to the end of life are the second largest contributor to the total LCC, where plant disposal for extraction component was considered. This value ranged from 1.71 € L^−1^ for the INN plant to 1.49 € L^−1^ for the CONV scenario. The operating costs of the extraction phase contributed on average of 10% to the total LCC for both scenarios. However, in the case of the INN plant, this value was equal to 1.14 € L^−1^, while in the CONV plant was 1.06 € L^−1^. The results in Table 7 show that the major cost contributors to the olive oil extraction cost were the fixed costs, representing on average 83% to the total for both systems. For the innovative technology, the fixed costs were greater, estimated at 0.95 € L^−1^, or 7.77% higher than conventional technology. This is largely due to the bigger costs of depreciation, insurance, and maintenance incurred for machinery and land investment because the innovative extraction plant with low oxidative impact reaches the larger initial investment costs.

The main cost hotspot within the variable costs were the inputs used to extract the olive oil, equivalent to 0.068 € L^−1^ in the INN scenario and 0.065 € L^−1^ in the CONV one. This is due to the electricity cost by machinery, which accounted for 38.18% and 37.53% of the costs, respectively.

As indicated in Table 8, the lowest profitability and investment feasibility were found for INN technology. As expected, the higher costs and the lesser extraction yield achieved in the innovative scenario led to a decrease in the DGM and NPV indicators as well as an increase in the DLCC indicator compared to the conventional one. However, the variations were not very high, with the DGM and NPV being 0.29% (5.14 € L^−1^ vs. 5.16 € L^−1^) and 3.12% (3.94 € L^−1^ vs. 4.06 € L^−1^) lower, respectively. The difference grows in terms of discounted costs, with an increase of 10.20% in the DLCC compared to the CONV scenario (1.37 € L^−1^ vs. 1.24 € L^−1^).

**Figure 6:**
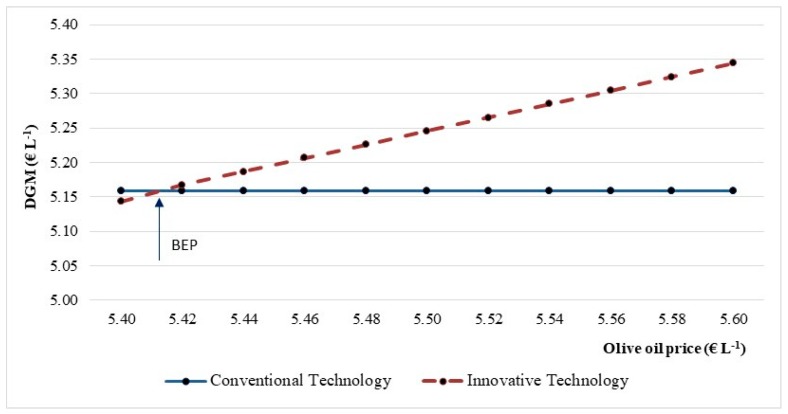
Break-even point (BEP) for the discounted gross margin (DGM) indicator in the function of the olive oil market price.

To explore how the economic performance of the innovative scenario could be improved compared to the conventional scenario, a sensitivity analysis was performed by changing the EVO oil selling price. This analysis was conducted by assuming a range between 5.40 and 5.60 € L^−1^, also to reflect the market price change. Moreover, the price increase assumed in this work is largely justified by the improvement in the quality of the EVOO obtained. As can be observed in Figure 6, an increase of 0.02 € L^−1^ in the olive oil price has positively affected the profitability of the innovative technology, increasing the DGM indicator by 0.38 p.p. and reaching a break-even price of 5.42 € L^−1^.

**Figure 7:**
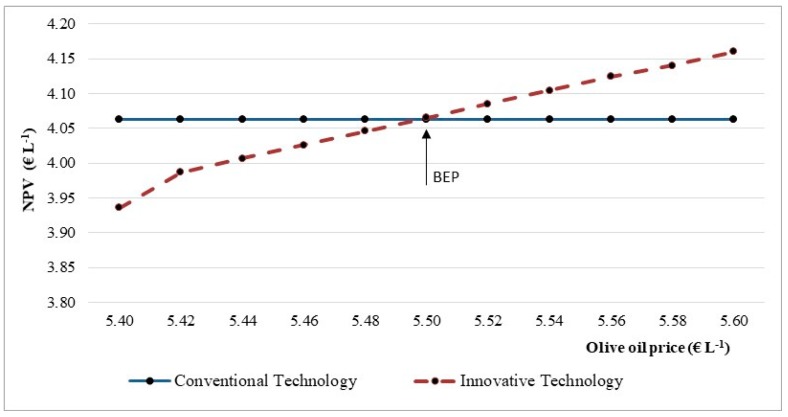
Break-even point (BEP) for the net present value (NPV) indicator in the function of the olive oil market price.

The results in Figure 7 indicated that an increase of 0.02 € L^−1^ in the olive oil price raised the NPV indicator by 0.50 p.p. The break-even price was reached at 5.50 € L^−1^. Therefore, these findings suggest that the larger the olive oil price, the higher the investment viability.

## 4. Discussion

The experimental technology with a low oxidative impact for EVOO extraction under study allowed heating the olive paste before malaxation and using a special decanter that avoids the final vertical centrifugation. The results of the experimental trials by the innovative plant showed a significant improvement of oil quality in terms of lesser peroxide content and higher chlorophylls, total polyphenols, and tocopherols content, as well as increased improvement of antioxidant activity, with respect to the conventional process. These findings are in general agreement with those obtained by Amirante et al. [15] and Esposto et al. [16], even if they worked under different experimental conditions and adopted different analytical methods for the quality evaluation of olive oil. Fadda et al. [14] also found that the lower oxidative stress exerted by innovative technology explains the lower peroxide content in the oils obtained, confirming our findings.

The economic results of the present study indicated that by applying a life cycle approach, the highest total costs were reached for innovative technology. The main contributor to the total LCC was the start-up stage, in which the highest cost was incurred for the investment in the innovative olive oil extraction machinery. This result involved high fixed costs related to the depreciation, insurance, and maintenance of the machinery. Within the variable costs, the electricity cost by machinery was most impacting. To the authors’ best knowledge, specific analyses that apply the LCC method to evaluate the economic performance of innovative extraction technologies are not implemented, so a comparison with previous papers becomes difficult.

Due to the highest LCC, innovative technology revealed the lowest profitability and investment feasibility than the conventional one. However, assuming an increase in the EVO oil selling price an improvement of its economic performance was reached. This demonstrates how the results could be affected by the methodological choices connected to the assumption of data invariance during the investment lifespan. These findings are consistent with results reported by De Gennaro et al. [49] and Falcone et al. [64].

Literature generally deals with the olive oil extraction stage as a single process, overlooking the impact of unitary operations in terms of environmental burdens [40]. The environmental assessment performed in this study showed that milling, malaxing, and centrifugal extraction were the main burdens contributing to the impact categories under investigation. These results are similar to those obtained by Guarino et al. [45], who compared two different olive oil extraction systems, though they analyzed the entire life cycle of the product, from olive cultivation to bottling. The innovative technology emphasized the impacts of the malaxation process, due to the increasing energy consumption. This might seem a contradiction since, as demonstrated by the results of the conventional technology assessment and as confirmed by other authors [45], the malaxation represents one of the hotspots of the olive oil extraction.

The innovative technology was also tested in order to design a quality-oriented process, not quantity-oriented. As also suggested by Ferrari et al. [72], it would be appropriate assessing the effects of quality on the environmental profile of products. In this direction, the present study evaluated the impacts of olive oil from the point of view of quality enhancement, by analyzing the real function of the innovative plant through the implementation of 1 mg of quality parameters as FU. The results showed the reduction of the impacts linked to the quality improvements. Moreover, optimization of the innovative plant aimed at increasing oil yields, as shown by the sensitivity analysis carried out, should lead to an improvement of the environmental profile in addition to the enhancement in quality already achieved.

The olive oil extraction experiments performed in this work can bring competitive advantages to the company, providing higher quality olive oil. A better quality of olive oil certainly involves many benefits from a commercial point of view and for consumer health, increasing its value added. As also argued by Roselli et al. [5], providing information on the healthy features of innovative products could be the most important marketing approach. This would allow companies to get a higher price on the market, thus increasing the investment profitability. Moreover, in line with conclusions drawn by Clodoveo et al. [73], we argue that optimization of energy use could reduce the operating costs during the milling and centrifugal extraction processes and thus, providing a good payback on capital investment. As already mentioned above, an energy saving could also lead to a reduction in environmental impacts. This result agrees with those obtained by Tsarouhas et al. [40], who point out that care should be taken to the efficient use of water and energy, as well as the reduction of air emissions and solid waste when moving towards an environmentally friendly production of olive oil.

The results of this research could be useful to raise both producer and consumer awareness of the technological advances in EVOO extraction. However, in future research new alternative extraction technologies should be explored in order to identify optimal solutions for increasing oil yields and reducing both operating costs and environmental impacts in a life cycle perspective.

## 5. Conclusions

The main purpose of this work was to explore the environmental and economic hotspots of an innovative olive milling plant located in Sardinia (South Italy), through the combination of LCA and LCC methodologies. A comparison with a conventional plant, which is still widespread in the EVOO extraction process, was also carried out. When the innovative system is used, a major improvement of oil quality compared to the conventional one was reached, although the lowest oil yields were attained. In particular, the advantage due to the lower oxidative stress exerted by the innovative technology has led to a significantly lower peroxide content. At the same way, a further quality enhancement was reached in terms of chlorophylls, total polyphenols, and tocopherols, and therefore as concerns the antioxidant activity.

As the environmental and economic findings are strictly affected by the production yield, greater environmental impacts and costs were achieved with the new extraction system. Thus, in terms of FU (1 L of EVO oil), the conventional plant was favored. However, as validated by the sensitivity analysis, an increase in the olive oil selling price and oil yields could lead to an improvement in the economic and environmental profile, respectively. These findings may be important for the sustainability evaluation of innovations in the olive oil industry.

## Figures and Tables

**Table 1:** Life cycle assessment (LCA) primary data inventory per scenario, input, and process phase.

	Conventional		Innovative	
	Electricity	Water	Electricity	Water
	kW L^−1^	m^3^ L^−1^	kW L^−1^	m^3^ L^−1^
Unloading olives	2.22 × 10^−3^		2.59 × 10^−3^	
Leaf removal and olive washing	1.18 × 10^−2^	4.92 × 10^−5^	5.75 × 10^−2^	5.75 × 10^−5^
Milling	4.39 × 10^−2^	9.85 × 10^−5^	4.46 × 10^−2^	1.15 × 10^−4^
Malaxing	1.02 × 10^−2^	9.85 × 10^−5^	2.86 × 10^−2^	1.15 × 10^−4^
Centrifugal extraction	4.04 × 10^−2^	2.46 × 10^−4^	3.14 × 10^−2^	2.87 × 10^−4^
Separation oil/water	9.02 × 10^−3^	1.23 × 10^−4^	2.37 × 10^−2^	1.44 × 10^−4^

LCA: Life cycle assessment.

**Table 2:** Impact categories considered in the study.

Impact Category	Abbreviation	Unit
Climate change	CC	kg CO_2_ eq
Ozone depletion	OD	kg CFC-11 eq
Human toxicity, non-cancer effects	HT noc	CTUh
Human toxicity, cancer effects	HT C	CTUh
Particulate matter	PM	kg PM2.5 eq
Photochemical ozone formation	POF	kg NMVOC eq
Acidification	AC	molc H+ eq
Terrestrial eutrophication	TE	molc N eq
Freshwater eutrophication	FE	kg P eq
Marine eutrophication	ME	kg N eq
Freshwater ecotoxicity	FT	CTUe
Water resource depletion	WD	m^3^ water eq
Mineral, fossil, and ren resource depletion	MFDR	kg Sb eq

**Table 3:** The main parameters for the economic analysis in the conventional and innovative scenarios.

Item	Unit	Amount
Electricity price	€ kWh^−1^	0.23
Current wage	€ h^−1^	7.30
Average price of EVO oil	€ L^−1^	5.40
Harvesting season	years	2017/2018
Life cycle	years	20
Discount rate	%	2

EVO: Extra virgin olive.

**Table 4:** Economic indicator description.

Indicator	Code	Formula	Unit
Discounted Gross Margin	DGM	$\frac{\sum_{j=1}^{n}\frac{TR}{{\left(1+r\right)}^{j}}-\frac{VC}{{\left(1+r\right)}^{j}}}{\sum_{j=1}^{n}TP}$	€ L^−1^
Net Present Value	NPV	$\frac{\sum_{j=0}^{n}\frac{B_{j}}{{\left(1+r\right)}^{j}}-\frac{C_{j}}{{\left(1+r\right)}^{j}}}{\sum_{j=1}^{n}TP}$	€ L^−1^
Discounted Life Cycle Costs	DLCC	$\frac{\sum_{j=1}^{n}IC+OP+DC}{\sum_{j=1}^{n}TP}$	€ L^−1^

Discounted gross margin (DGM), net present value (NPV), and discounted life cycle costs (DLCC).

**Table 5:** Influence of extraction technology on extraction yield and main quality parameters of Bosana extra-virgin olive oils.

Technology	Yield	Free Acidity (% Oleic Acid)	Peroxide Value (meq O^2^ kg^−1^ Oil)	Chlorophills (mg Pheophytin A kg^−1^ Oil)	Total Polyphenols (mg Gallic Acid kg^−1^ Oil)	Total Tocopherols (mg α-Tocopherol kg^−1^ Oil)	Antioxidant Activity (EC50 mg g^−1^)
Conventional	20.2 ^a^	0.17 ^a^	12.2 ^a^	26.8 ^b^	412.9 ^b^	307.3 ^b^	39.19 ^a^
Innovative	17.3 ^b^	0.17 ^a^	6.9 ^b^	30.3 ^a^	487.3 ^a^	390.9 ^a^	30.23 ^b^

Data followed by different letters for each column are significantly different by Tukey’s Test at *p* ≤ 0.01.

**Table 6:** Characterization of impacts with ILCD 2011 midpoint method.

Impact Category	Unit	Conventional	Innovative	Variation I/C
CC	kg CO_2_ eq	9.03 × 10^−2^	9.46 × 10^−2^	+4.80%
OD	kg CFC-11 eq	6.92 × 10^−9^	7.13 × 10^−9^	+2.95%
HT noc	CTUh	6.62 × 10^−9^	6.93 × 10^−9^	+4.76%
HT C	CTUh	2.11 × 10^−9^	2.22 × 10^−9^	+5.00%
PM	kg PM2.5 eq	2.98 × 10^−5^	3.10 × 10^−5^	+4.13%
POF	kg NMVOC eq	2.08 × 10^−4^	2.15 × 10^−4^	+3.37%
AC	molc H+ eq	4.38 × 10^−4^	4.52 × 10^−4^	+3.28%
TE	molc N eq	7.14 × 10^−4^	7.39 × 10^-4^	+3.46%
FE	kg P eq	1.61 × 10^−5^	1.68 × 10^−5^	+3.91%
ME	kg N eq	8.44 × 10^−5^	8.95 × 10^−5^	+5.98%
FT	CTUe	1.91 × 10^−1^	2.03 × 10^−1^	+5.97%
WD	m^3^ water eq	4.44 × 10^−4^	4.57 × 10^−4^	+3.04%
MFDR	kg Sb eq	1.52 × 10^−7^	1.68 × 10^−7^	+10.18%

Notes: CC: Climate change; OD: Ozone depletion; HT noc: Human toxicity, non- cancer effects; HTC: Human toxicity, cancer effects; PM: Particulate matter; POF: Photochemical ozone formation; AC: Acidification; TE: Terrestrial eutrophication; FE: Freshwater eutrophication; ME: Marine eutrophication; FT: Freshwater ecotoxicity; WD: Water resource depletion; and MFDR: Mineral, fossil, and ren resource depletion.

**Table 7:** Olive oil extraction cost of the analyzed scenarios.

Cost Item	Conventional Technology	Innovative Technology
	(€ L^−1^)	(€ L^−1^)
Total variable costs (A)	0.18	0.19
Input costs for olive oil extraction	0.065	0.068
Human labor cost	0.10	0.12
Interests on advance capital	0.006	0.007
Total fixed costs (B)	0.88	0.95
Machinery and land investments ownership costs	0.40	0.45
Land rent	0.275	0.279
Interests on capital goods	0.05	0.06
Taxes	0.016	0.019
Administration overheads	0.14	0.14
Total extraction cost (A+B)	1.06	1.14

**Table 8:** Economic impact results (€ L^−1^).

Economic Indicators	Life Cycle Results	
	Conventional Technology	Innovative Technology
	(€ L^−1^)	(€ L^−1^)
Discounted Gross Margin (DGM)	5.16	5.14
Net Present Value (NPV)	4.06	3.94
Discounted Life Cycle Costs (DLCC)	1.24	1.37
